# Baseline magnetic resonance imaging findings associated with short-term clinical outcomes after intraarticular administration of autologous adipose-derived stem cells for knee osteoarthritis

**DOI:** 10.1016/j.reth.2024.12.012

**Published:** 2024-12-26

**Authors:** Ryota Yamagami, Tomohiro Terao, Taro Kasai, Hisatoshi Ishikura, Masaki Hatano, Junya Higuchi, Shuichi Yoshida, Yusuke Arino, Ryo Murakami, Masashi Sato, Yuji Maenohara, Yuma Makii, Tokio Matsuzaki, Keita Inoue, Shinsaku Tsuji, Sakae Tanaka, Taku Saito

**Affiliations:** a Department of Orthopaedic Surgery, Faculty of Medicine, The University of Tokyo, Tokyo, Japan; b Ochanomizu Cell Clinic, Tokyo, Japan; c Yakuendai Rehabilitation Hospital, Chiba, Japan; d Avenue Cell Clinic, Tokyo, Japan; e CPC Corporation, Tokyo, Japan

**Keywords:** Adipose-derived stem cell, Bone marrow lesion, Meniscus extrusion, Magnetic resonance imaging, MRI osteoarthritis knee score, Knee osteoarthritis

## Abstract

**Introduction:**

This study aimed to determine the association between the baseline magnetic resonance imaging (MRI) findings and clinical outcomes after articular injection of adipose-derived mesenchymal stem cells (ASCs) for knee osteoarthritis (KOA).

**Methods:**

This retrospective study included 149 patients with varus-type KOA treated with a single intraarticular ASC injection. All patients underwent a MRI evaluation before treatment. Patients were categorized following the MRI Osteoarthritis Knee Score (MOAKS) system cartilage score into the mild, moderate, or severe KOA groups. Additionally, joint effusion and synovitis, bone marrow lesions (BMLs), and meniscal extrusions were graded with the MOAKS. Knee Osteoarthritis Outcome Score (KOOS) was obtained at baseline, 1-, 3-, 6-, and 12-month posttreatment. The responder rate in the Outcome Measures in Arthritis Clinical Trials-Osteoarthritis Research Society International was assessed with the KOOS. Multivariate logistic regression analyses were conducted to determine factors associated with the responder rate.

**Results:**

All KOOS subscales significantly enhanced with the greatest improvement from baseline to 6 months which plateaued between 6 and 12 months. The responder rate was 65.4 % in the mild/moderate KOA compared to 35.2 % in the severe KOA at 12 months. Lower OA grade (odds ratio [OR]: 0.52; 95 % confidence interval (CI): 0.31–0.88; *P* = 0.015), smaller BMLs in medial femoral condyle (OR: 0.36; 95 % CI: 0.14–0.94; *P* = 0.037), and less meniscal extrusion (OR: 0.31; 95 % CI: 0.11–0.89; *P* = 0.029) were associated with higher responder rate at 6 months in multivariable logistic regression analysis. The factors associated with higher responder rate at 12 months included lower OA grade (OR: 0.42; 95 % CI: 0.25–0.73; *P* = 0.002) and younger age (OR: 1.04; 95 % CI: 1.00–1.08; *P* = 0.042).

**Conclusions:**

ASC treatment for KOA enhanced short-term clinical outcomes. MRI findings, including cartilage lesions, BMLs, and meniscal extrusion, were associated with responder rate, helping physicians identify which patients may benefit from this therapy.

## Highlights

• Adipose-derived stem cell (ASC) injection can benefit knee osteoarthritis (OA).
• Associations between baseline MRI findings and clinical outcomes were investigated.
• Patients with mild to moderate OA respond better to ASCs than those with severe OA.
• Meniscus extrusion and bone marrow lesions were linked with lower efficacy rate.
• MRI-based evaluations help predict the effectiveness of ASC therapy for knee OA.

## 1 Introduction

Knee osteoarthritis (KOA) is a degenerative deformity that causes chronic pain and functional limitations and reduces patients’ quality of life (QOL) [1]. Additionally, its number has been increasing in recent years in many countries [2,3]. The conventional conservative treatment of KOA involves patient education (e.g., weight loss), physical therapy (e.g., lower limb muscle strengthening), and oral, local, and intraarticular pharmacological therapies (e.g., nonsteroidal anti-inflammatory drugs and hyaluronic acid injections) [[4], [5], [6], [7]]. Patients who achieve no pain relief after receiving adequate conservative treatment are indicated for osteotomy or arthroplasty [8].

Recently, biologic therapies such as intraarticular injections of platelet-rich plasma, stromal vascular fragments and mesenchymal stem cells (MSCs) for the treatment of KOA have gained attention. Among these, cultured MSCs offer advantages, including high consistency in controlling cell numbers for therapeutic doses, compared to the other two methods, whose regenerative potential may vary depending on tissue source and application method [[9], [10], [11]]. In 2010, several case reports and case series [12,13] and randomized controlled trials [14] of intraarticular MSC administration for human KOA were published, all of which revealed significant improvements in pain scores on the visual analog scale and the Western Ontario MacMaster University Osteoarthritis Index score. Additionally, a recent systematic review [15] indicated that intraarticular adipose-derived stem cell (ASC) administration was effective in suppressing pain for 6–12 months after administration. Conversely, evidence on the mechanism of pain suppression and factors that are influential on the clinical outcomes of ASCs remains insufficient.

Some studies [16,17] have demonstrated that KOA severity was associated with treatment outcomes, and these studies usually utilize the Kellgren–Lawrence (KL) classification based on knee radiographs. Conversely, magnetic resonance imaging (MRI) detects more detailed KOA information, including cartilage defects, synovitis, and meniscal conditions [18,19]. However, the association between more detailed intraarticular findings by MRI and outcomes after intraarticular ASC administration remains unclear.

This study aimed to determine the association between short-term clinical outcomes after intraarticular administration of autologous ASCs for KOA and MRI findings before administration. We hypothesized that findings, such as cartilage lesions, joint effusion, meniscal conditions, and bone marrow lesions (BMLs), would affect clinical outcomes.

## 2 Materials and methods

### 2.1 ASC therapy for KOA

We have been administering intraarticular injection of cultured autologous ASCs for KOA at the Ochanomizu Cell Clinic since August 2019. Patients who have not received conservative treatment before the administration are instructed to first receive conservative treatment, and those who have not achieved sufficient improvement in symptoms despite standard conservative treatment are included in the administration. Patients who met the following criteria were unable to undergo this treatment: hypersensitivity to anesthetics for adipose collection or substances for the manufacturing process, such as egg protein, an allergic reaction to penicillin, streptomycin, or amphotericin B for cell culture, positive pathogenic microbiological tests, including hepatitis B virus, hepatitis C virus, human immunodeficiency virus, and syphilis, age of <20 years, pregnant or lactating women, complications with severe trauma, poor understanding of the therapy, and abnormal prothrombin time or activated partial thromboplastin time before treatment.

### 2.2 ASC preparation

Abdominal subcutaneous adipose was transcutaneously collected under local anesthesia for ASC preparation [20]. The collected tissue was immediately transferred to the cell processing center at the CPC Corporation. Cells were isolated from the tissue with an unwoven fabric and cultured in the optimized medium with 1%–4% autologous serum for 3–4 weeks up to approximately 1 × 10^8^ cells at 37 °C with 5 % CO_2_ [21].

### 2.3 Intraarticular injection of ASCs

Approximately 1 × 10^8^ ASCs were injected into the medial tibiofemoral joint space when the main lesion was located in the medial compartment, and into the lateral tibiofemoral joint space when it was in the lateral compartment with a 23 G needle after completing the culturing process. We did not limit the joint motion or daily activity after the ASC therapy.

### 2.4 Patient data collection

The Ethics Committee of the University of Tokyo approved this study, and patients provided informed consented to participate in the study.

This study reviewed retrospectively 174 patients who received intraarticular ASCs for KOA with varus deformity from August 2019 to May 2022. All these patients were followed up for at least 12 months after treatment. Exclusion criteria were (1) patients without necessary MRI sequences for evaluation as described below within 3 months before treatment (n = 11), (2) those with a main lesion in the patellofemoral joint of KL grade of >3 (n = 6), (3) those with concomitant idiopathic knee osteonecrosis (n = 4), and (4) those who received multiple ASC administrations (n = 4). Hence, the final analysis set included the remaining 149 patients. The group consisted of 51 males and 98 females, with a mean age of 67.0 years (range, 37–93 years) and a mean body mass index of 24.5 kg/m^2^ (range, 18.0–33.8 kg/m^2^).

### 2.5 MRI finding evaluation

Several subscales of the MRI Osteoarthritis Knee Score (MOAKS) [18] were adopted before ASC administration to assess the cartilage lesions, synovitis, BMLs, and meniscus lesions, which were indicated in previous studies [[22], [23], [24]] as influential factors on symptoms and pain of patients with KOA. The MOAKS is a quantitative scoring system for KOA used in many previous reports, with higher scores in more advanced OA.

**Fig. 1:**
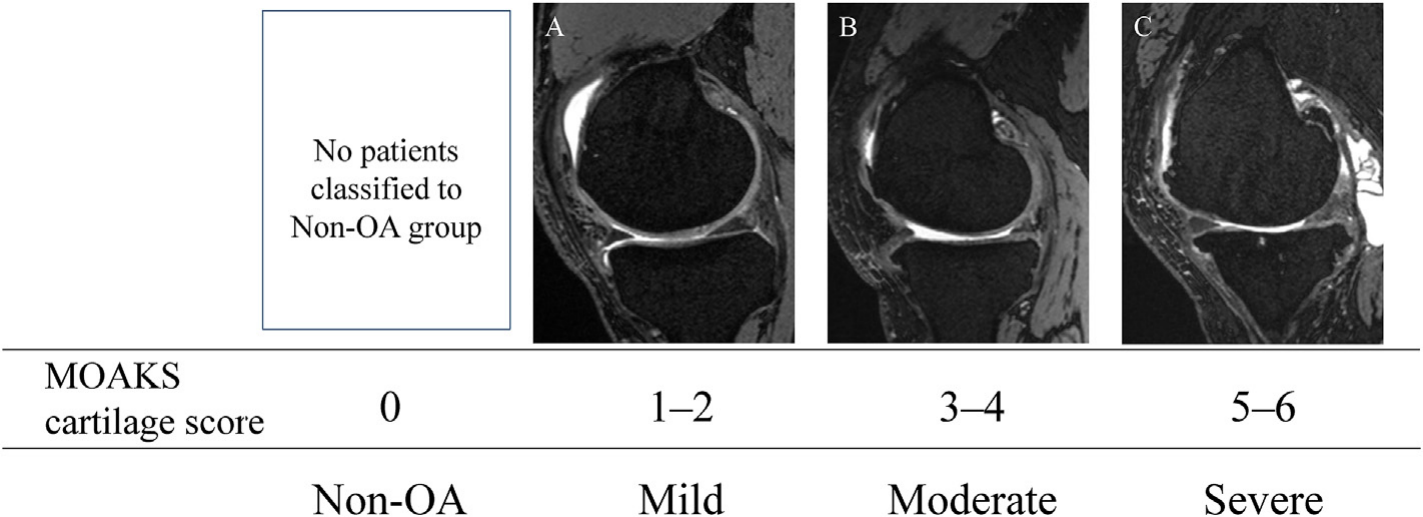
Knee osteoarthritis grading based on MOAKS cartilage score in the medial compartment of the knee. Typical cases are presented, including mild osteoarthritis (A), moderate osteoarthritis (B), and severe osteoarthritis (C). OA: osteoarthritis.

First, KOA grading was conducted based on the MOAKS cartilage score using MRI with standard T2/intermediate-weighted (IW) or proton density (PD)-weighted fat-saturated turbo-spin-echo sequences [18]. This study that analyzes patients with KOA with varus deformity evaluated the medial femoral condyle (MFC) and medial tibial condyle (MTP) as regions of interest and identified scores in each region from 0 to 6 points based on the size and depth of cartilage damage (Table 1). The higher score of MFC and MTP was then utilized as the final score of the patient; 0 points were classified as non-OA group, 1–2 points as mild KOA group, 3–4 points as moderate KOA group, and 5–6 points as severe KOA group (Fig. 1) as previously reported [25]. In this study, no patients were classified into Non-OA group.

**Table 1:** MOAKS grading for cartilage.

Findings on MR images	MOAKS score for % full thickness	MOAKS score for size of cartilage lesion	MOAKS cartilage score^a^
Normal	0	0	0
1%–10 % of the cartilage area damaged No full-thickness cartilage loss	0	1	1
1%–10 % of the cartilage area damaged 1%–10 % full-thickness cartilage loss	1	1	2
10%–75 % of the cartilage area damaged No full-thickness cartilage loss	0	2	2
10%–75 % of the cartilage area damaged 1%–10 % full-thickness cartilage loss	1	2	3
10%–75 % of the cartilage area damaged 10%–75 % full-thickness cartilage loss	2	2	4
>75 % of the cartilage area damaged No full-thickness cartilage loss	0	3	3
>75 % of the cartilage area damaged 1%–10 % full-thickness cartilage loss	1	3	4
>75 % of the cartilage area damaged 10%–75 % full-thickness cartilage loss	2	3	5
>75 % of the cartilage area damaged >75 % full-thickness cartilage loss	3	3	6

MR: magnetic resonance.

a MOAKS cartilage score is the sum of the MOAKS score for % full thickness and the MOAKS score for the size of cartilage lesions.

Second, the degree of joint effusion and synovitis was graded between grades 0 and 3 based on the MOAKS effusion-synovitis score. T2/IW or PD-weighted fat-saturated images of the axial plane were utilized for these evaluations. Grades 0, 1, 2, and 3 indicated physiologic, small (fluid continuous in the retropatellar space), medium (with slight convexity of the suprapatellar bursa), and large (evidence of capsular distention) amounts, respectively [18].

Third, BMLs were evaluated on MRI with proton density (PD)-weighted fat-saturated or short T1 inversion recovery sequences in the coronal planes using the MOAKS BML score for MFC and MTP. The BML size was evaluated on coronal images of the central region (defined as the anteroposterior mid-one third of the femoral and tibial condyle) and graded as follows: grade 0: none, grade 1: <33 % of regional volume, grade 2: 33%–66 % of regional volume, and grade 3: >66 % of regional volume [18].

Lastly, medial meniscus (MM) extrusion was assessed on the mid-slices of the coronal plane of T2-weighted or PD fat-saturated images. The distance of MM extrusion from the medial margin of the medial tibial condyle, excluding osteophytes, was measured and classified from grade 0 to grade 3 as < 2 mm, 2–2.9 mm, 3–4.9 mm, and >5 mm, respectively [18].

To ensure intra- and inter-observer reproducibility for the MRI evaluations, a single researcher (RY) evaluated MRI findings twice, with a minimum three-month interval, in 20 randomly selected patients from the studied group. A second examiner (RM) independently performed the evaluations for the same patient group. Cohen's kappa, calculated to assess both intra- and inter-rater reliability based on these measurements, was >0.80 for all categories, indicating excellent agreement.

### 2.6 Clinical outcome evaluation

**Fig. 2:**
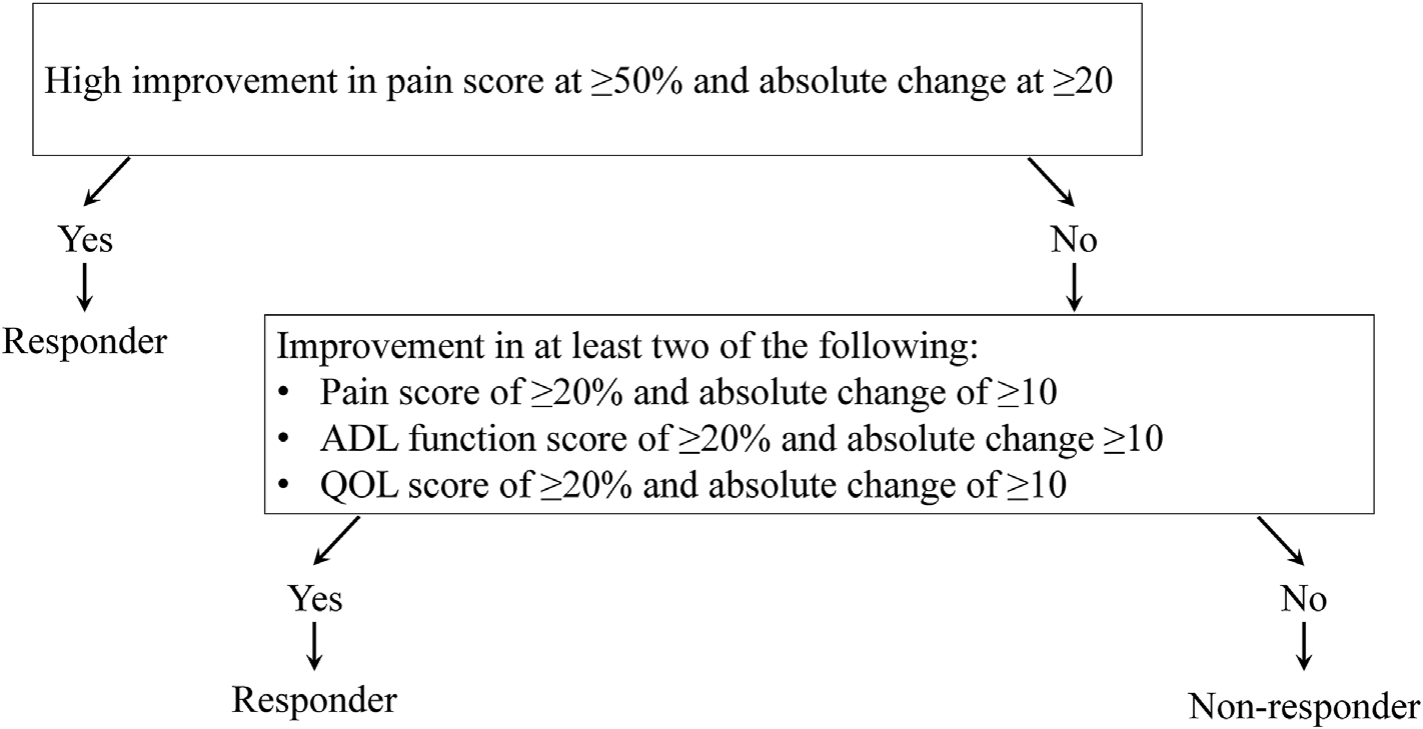
The responder criteria of Outcome Measures in Arthritis Clinical Trials-Osteoarthritis Research Society International (OMERACT-OARSI).

Patient-reported outcome measures (PROMs) were assessed with Knee Osteoarthritis Outcome Score (KOOS) [26] at pretreatment, 1-, 3-, 6-, and 12-month posttreatment. KOOS has five subscales, namely pain, symptoms, activities of daily living (ADL) for physical function, sport recreation function, and knee-related QOL. Each subscale is scored separately on a scale from 0 to 100, indicating extreme knee problems and no knee problems, respectively. Subsequently, KOOS total score was calculated on a scale from 0 to 100 based on the number of questionnaire items of five subscales. Additionally, the Outcome Measures in Arthritis Clinical Trials-Osteoarthritis Research Society International (OMERACT-OARSI) criteria [27] were assessed with KOOS subscales of pain, ADL function, and QOL subscales (Fig. 2).

### 2.7 Statistical analysis

All statistical analyses were conducted with BellCurve for Excel version 4.07 (Social Survey Research Information Co., Ltd., Tokyo, Japan). A one-way repeated measures analysis of variance (ANOVA) and post-hoc pairwise comparison (Tukey test) was utilized to analyze the changes in KOOS subscales and total scores over time. A two-way repeated measures ANOVA and post-hoc pairwise comparison (Turkey test) was employed to compare the KOOS total scores of three OA groups at each time point. The patients' background factors (age, sex, and body mass index [BMI]) and the MRI findings were compared between the responder and the nonresponder of the OMERACT-OARSI criteria using Student's *t*-test for continuous variables and Chi-square test with post-hoc Bonferroni comparisons for nominal variables. Multivariable logistic regression analysis was conducted to analyze factors associated with the responders at 6 and 12 months following ASC injection calculating odds ratio (OR) and 95 % confidence intervals (CI). Patients' age, sex, and BMI were included in the multivariable models in addition to the MRI findings. A priori power analysis for one-way repeated measures ANOVA was conducted with G∗Power (version 3.1.9.7, Heinrich Heine University, Düsseldorf, Germany) before this study. The alpha set, power, and effect size of 0.05, 0.8, and 0.25, respectively, required 21 patients. A *P*-value of <0.05 indicated statistical significance. Data were presented as means and standard deviations for continuous variables and frequency counts and percentages for nominal variables.

## 3 Results

**Fig. 3:**
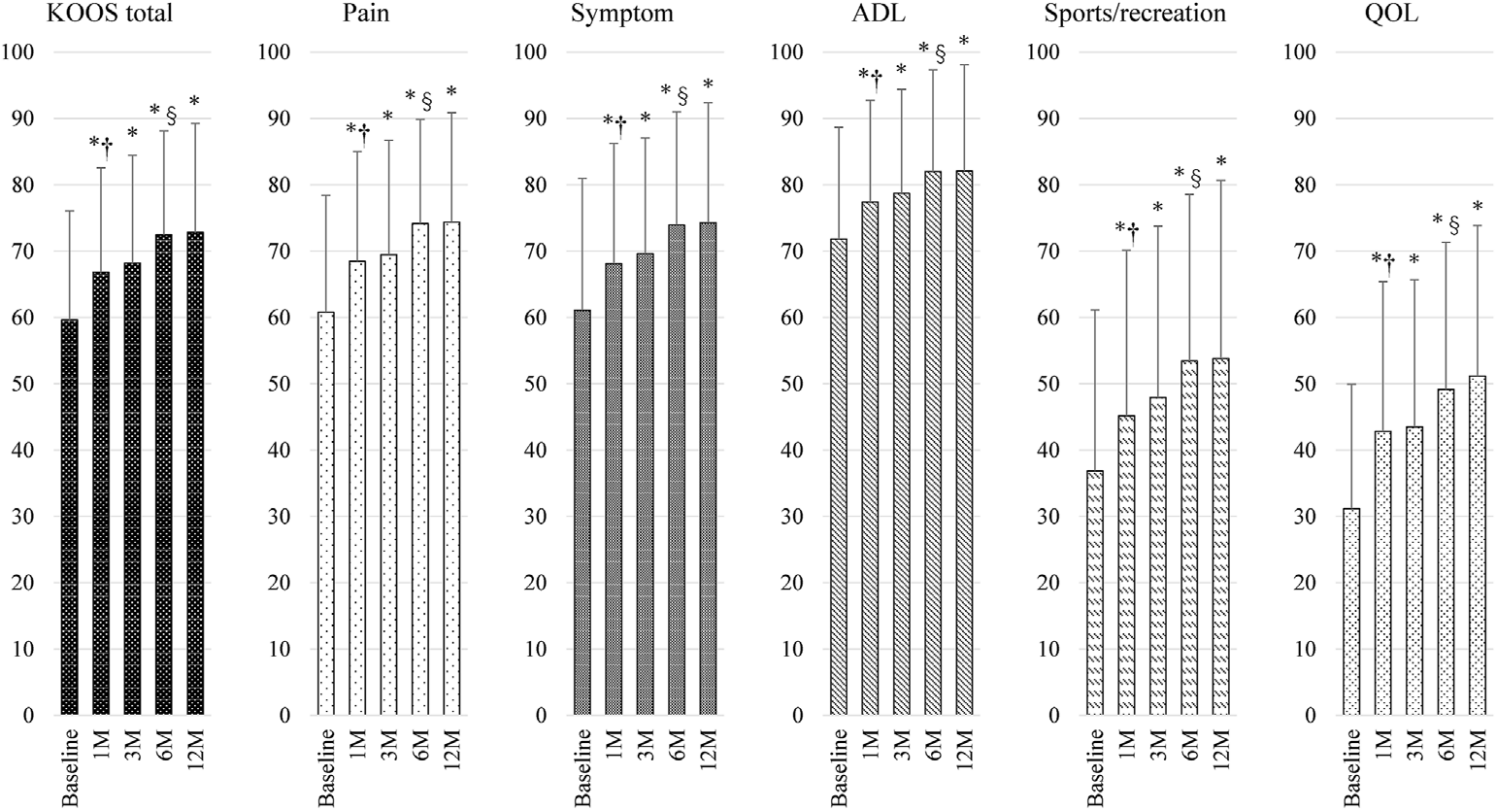
Chronological change of KOOS total and subscales in the analysis of the entire patients. A one-way repeated measures analysis of variance and post-hoc pairwise comparison (Tukey test) was conducted. Graphs show the mean and standard deviation (SD). ∗, *P* < 0.05 compared to the baseline. †, *P* < 0.05 compared between scores at baseline and those at 1 month. §, *P* < 0.05 compared between scores at 3 months and those at 6 months. KOOS: knee osteoarthritis outcome score; ADL: activity of daily living; QOL: quality of life; SD: standard deviation; M: month.

**Fig. 4:**
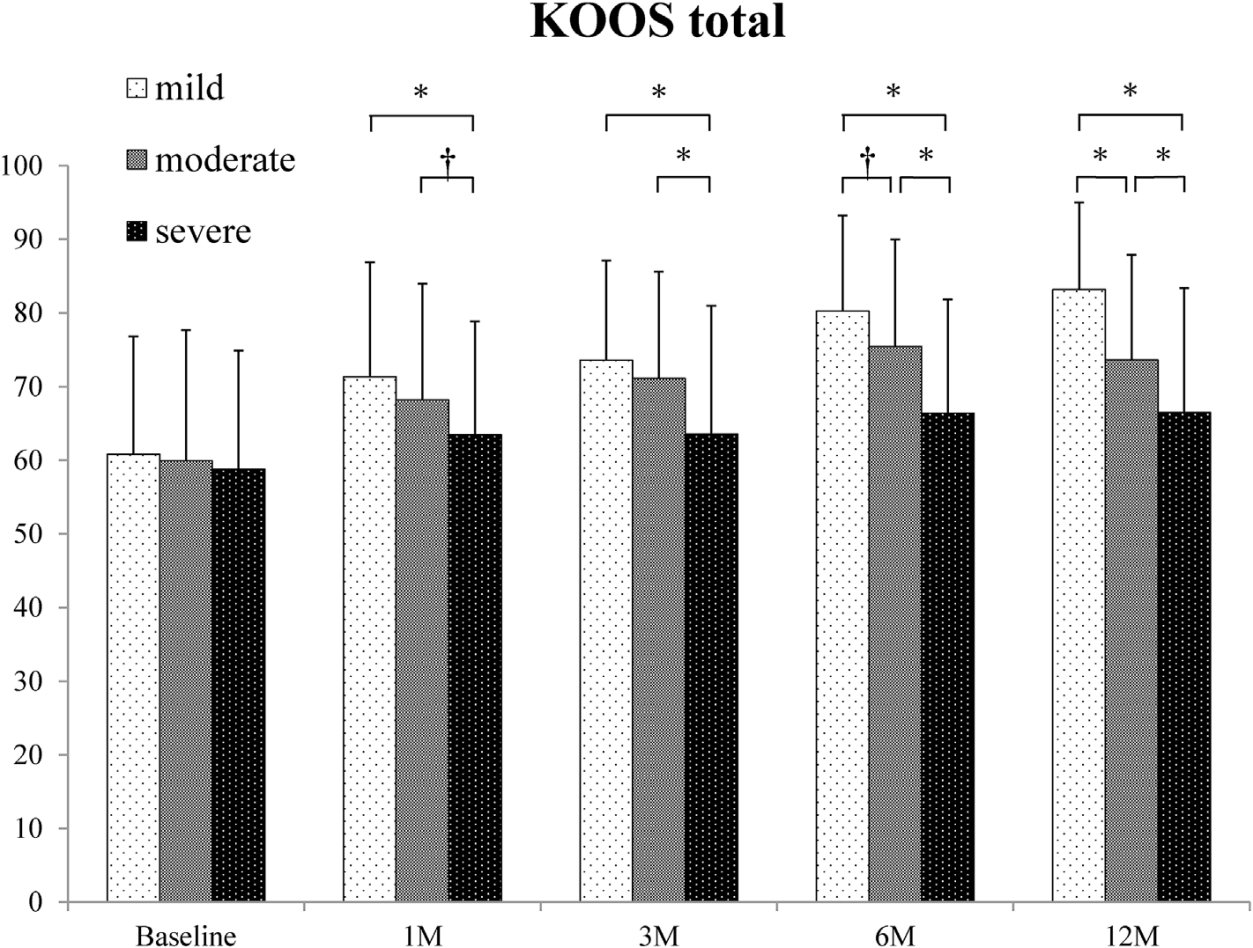
Chronological change of KOOS total score by OA grade. A two-way repeated measures analysis of variance and post-hoc pairwise comparison (Tukey test) was conducted to compare KOOS total scores of three OA grade groups at each time point. Graphs show the mean and SD. The baseline score was not significantly different among groups. Mild- and moderate-grade OA had better scores than severe-grade OA in 1 month and 3 months after the ASC injection. Mild-grade OA had better scores than moderate- and severe-grade OA after 6 months. ∗, *P* < 0.001; †, *P* < 0.01.

The analysis of the entire patients revealed that the KOOS total score and each of its subscales, namely, pain, symptoms, ADL, sports/recreation, and QOL, demonstrated significant improvement at 1, 3, 6, and 12 months after the ASC injection compared with that at the baseline (Fig. 3). Moreover, a significant stepwise improvement was found from the baseline to 1 month and from 3 months to 6 months posttreatment, but with no significant change from 1 month to 3 months and from 6 months to 12 months posttreatment in both total scores and all subscales, respectively. A subanalysis exhibited that KOOS total score by KOA grades based on MOAKS cartilage score was significantly better in mild- and moderate-grade KOA than severe-grade KOA at 1 month and 3 months and was significantly better in mild-grade KOA than both moderate- and severe-grade OA after 6 months, whereas baseline score was not significantly different among groups (Fig. 4).

The responder rate following the OMERACT-OARSI criteria at each time point was calculated. The responder rate gradually increased from 29.5 % at 1 month and 42.3 % at 3 months to 51.7 % at 6 months after the injection; however, almost no change was observed up to 51.0 % at 12 months posttreatment.

The comparison between the responder and the nonresponder at 6 and 12 months after ASC injection indicated no significant differences in age (6 months: 67.3 ± 9.4 vs. 66.6 ± 10.7, *P* = 0.678; 12 months: 67.7 ± 9.4 vs. 66.2 ± 10.7, *P* = 0.366) and BMI (6 months: 25.0 ± 3.6 kg/m^2^ vs. 24.0 ± 3.5 kg/m^2^, *P* = 0.091; 12 months: 24.9 ± 3.6 kg/m^2^ vs. 24.1 ± 3.5 kg/m^2^, *P* = 0.198). Table 2 presents the proportion of the responders of the OMERCT-OARSI criteria at 6 and 12 months after ASC injection based on the patient's sex and MRI findings. Higher proportions of the responders were observed in patients with lower grades of KOA, and BML in MFC and MM extrusion at both 6 and 12 months posttreatment. Specifically, the responder rate of the total mild and moderate KOA group at 12 months was 65.4 % (51of 78), whereas that of the severe KOA group was 35.2 % (25 of 71).

**Table 2:** Baseline MRI findings and proportion of the responder at 6 and 12 months after ASC injection.

		Total n	Responder at 6 months n (%)	*P* value^a^	Responder at 12months n (%)	*P* value^a^
Overall		149	77 (51.7)		76 (51.0)	
Sex				0.521		0.867
	Male	51	24 (47.1)		27 (52.9)	
	Female	98	53 (54.1)		49 (50.0)	
					
MRI finding grade						
Cartilage (OA grade)				<0.001		<0.001
	Mild OA	40	28 (70.0)	b	30 (75.0)	b
	Moderate OA	38	26 (68.4)	b	21 (55.3)	
	Severe OA	71	23 (32.4)	c	25 (35.2)	c
Effusion and synovitis				0.164		0.698
	Grade 0	16	4 (25.0)		6 (37.5)	
	Grade 1	64	35 (54.7)		33 (51.6)	
	Grade 2	42	23 (54.8)		22 (52.4)	
	Grade 3	27	15 (55.6)		15 (55.6)	
Bone marrow lesion in MFC				<0.001		0.021
	Grade 0	49	32 (65.3)	b	29 (59.2)	
	Grade 1	38	24 (63.2)		24 (63.2)	
	Grade 2	41	10 (24.4)	c	13 (31.7)	c
	Grade 3	21	9 (42.8)		10 (47.6)	
Bone marrow lesion in MTP				0.751		0.898
	Grade 0	61	30 (49.2)		31 (50.8)	
	Grade 1	34	20 (58.8)		19 (55.9)	
	Grade 2	30	14 (46.7)		15 (50.0)	
	Grade 3	24	13 (54.2)		11 (45.8)	
MM extrusion				<0.001		0.006
	Grade 0	19	10 (52.6)		8 (42.1)	
	Grade 1	27	24 (88.9)	b	22 (81.5)	b
	Grade 2	33	12 (36.4)	c	15 (45.5)	
	Grade 3	70	31 (44.3)		31 (44.3)	

ASC: adipose-derived stem cell; OA: osteoarthritis; NA: not applicable; MFC: medial femoral condyle; MTP: medial tibial condyle; MM: medial meniscus.

a Responder vs non-Responder analyzed using Chi-square test.

b Significantly higher ratio in Responders (*P*-value <0.05) determined by post-hoc Bonferroni comparisons between Responders and Non-Responders.

c Significantly lower ratio in Responders (*P*-value <0.05) determined by post-hoc Bonferroni comparisons between Responders and Non-Responders.

The multivariable logistic regression analysis revealed that the efficacy rate at 6 months after ASC injection was significantly higher in lower OA grade (OR: 0.52; 95 % CI: 0.31–0.88; *P* = 0.015), MFC BML grade (OR: 0.36; 95 % CI: 0.14–0.94; *P* = 0.037), and MM extrusion grade (OR: 0.31; 95 % CI: 0.11–0.89; *P* = 0.029). Moreover, the efficacy rate at 12 months was significantly higher in lower OA grade (OR: 0.42; 95 % CI: 0.25–0.73; *P* = 0.002) and younger patients (OR: 1.04; 95 % CI: 1.00–1.08; *P* = 0.042).

## 4 Discussion

This study revealed several important results. First, intraarticular ASC administration for KOA significantly improved PROMs measured by KOOS at 6 months after administration, and these improvements were maintained until 12 months posttreatment. Additionally, the KOOS total score was significantly better in mild and moderate KOA than in severe KOA at every time point. Second, the responder rate was higher in patients with lower grades of KOA at both 6 and 12 months posttreatment. Additionally, other MRI findings, such as lower grade of BML of MFC and MM extrusion, were independently associated with higher responder rates at 6 months after ASC injection. To the best of our knowledge, this is the first paper that revealed the association between the baseline MRI findings and the clinical results after the ASC treatment for KOA.

This study revealed that intraarticular administration of ASC for KOA demonstrated lower efficacy in the severe OA group with more advanced lesions assessed by MOAKS cartilage score, compared to the mild or moderate OA group. Reportedly, the OA grading used in this study correlates well with the KL classification by plain X-ray [25]. Therefore, the results of this study can be translated into X-ray evaluation, that is, X-ray-based KL classification may predict the outcome of ACS. Kuwasawa et al. [17] compared the efficacy rate in OMERACT-OARSI criteria between the patients with severe (KL grade of 4) and mild (KL grade of 2/3) KOA in their retrospective observational study and revealed the increased efficacy rate of the mild KOA group over time up to 64 % at 6 months after intraarticular injection of ASC. Although the cell number administered in knee joints in our study was larger than in Kuwasawa's study, our results indicated that the efficacy rate of mild/moderate KOA was almost maintained from 69.2 % at 6 months to 65.4 % at 12 months. Conversely, the efficacy rate of the severe KOA group was only 35.2 % at 12 months. We did not compare the results of patients with KL grade of 4 with those who underwent knee arthroplasty, following the previous report by Weber et al. [28] showing an 86.1 % OMERACT-OARSI effective rate for total knee arthroplasty 1 year postoperatively, but physicians need to discuss the limitation of this therapy compared to knee arthroplasty in terms of patients who request ASC therapy for advanced KOA.

Moreover, this study revealed BML in MFC and MM extrusion as independent factors associated with the clinical outcomes after intraarticular ASC administration. These factors have been reported to be risk factors for KOA itself and to be related to KOA progression and pain. Guermazi et al. [23] revealed that meniscal extrusion assessed by MRI-based quantitative evaluation was associated with the advancement of cartilage loss in their longitudinal observational study. Moreover, Tanamas et al. [29] demonstrated that the severity of BMLs was negatively associated with cartilage loss over 2 years and positively associated with the risk of knee replacement over 4 years. Our results indicated that these prognostic factors in KOA affect outcomes after ASC injection. On the other hand, the association between baseline MRI findings and posttreatment outcomes which this study revealed will help physicians in predicting efficacy before ASC administration to some degree. Physicians had better identify whether ASC therapy will be beneficial for patients based on not only plane X-ray but also MRI before injection.

While this study demonstrates positive results, the underlying molecular mechanisms remain unclear [30,31]. Beyond their proliferation and multipotent differentiation capacities, recent attention has focused on the immunomodulatory, anti-inflammatory, and tissue-repairing effects of MSCs, which likely mediate their beneficial effects on OA pathophysiology. Clinical studies have reported symptoms and pain relief in OA patients following intraarticular MSC injections. However, significant structural changes, such as cartilage or meniscus regeneration, remain unproven [32]. Thus, the benefits of MSC therapy for OA may primarily derive from anti-inflammatory effects rather than tissue-repair. This assumption is consistent with our finding that intraarticular administration of ASC was more effective in patients with lower-grade OA or mild MM extrusion. However, the effectiveness observed in some advanced OA cases suggests the involvement of additional, as yet unidentified, pathways. Further research is warranted to elucidate these mechanisms.

The present study has some limitations despite meaningful results. First, a control group was not included in this study. While ASC injections were only provided to patients with persistent pain despite conservative treatment, future comparative studies are necessary to further validate the efficacy of ASC injections. Second, the follow-up period was relatively short. Evidence for middle-to long-term clinical outcomes after ASC therapy remains lacking; thus, the analysis of the long-term follow-up period will be necessary. Third, this study solely analyzed varus-type medial KOA. Therefore, the results of the present study cannot be applied to KOA in which the main lesion is in the lateral compartment or the patellofemoral joint. Fourth, this study did not investigate long leg alignment. A varus alignment is a factor that increases loading of the medial compartment and will be estimated to affect post-ASC outcomes, but full-length plain X-rays of the lower extremity were not taken in this study due to facility restrictions. Similarly, KL grading could not be evaluated for the same reason. Finally, posttreatment MRI was not assessed. Future studies are warranted to reveal how the findings seen on baseline MRI changed after treatment may be related to the clinical outcomes after ASC treatment.

## 5 Conclusions

In conclusion, intraarticular ASC administration for KOA improved PROMs in the short term. The baseline MRI findings, such as more severe cartilage lesion (higher KOA grade), BML size in MFC, and MM extrusion quantitatively assessed with MOAKS, were associated with the lower responder rate by OMERACT-OARSI criteria after intraarticular ASC administration. These facts may help physicians predict which patients will benefit from ASCs.

## Informed consent

The patients signed written informed consent. All data were handled under the declaration of Helsinki.

## Authors’ contributions

RY conceived and designed the study, collected and analyzed the data, and prepared the draft of the manuscript. TT, TK, HI, MH, JH, SY, YA, RM, MS, YujM, YumM, and TM conducted the ASC therapy and collected data. KI and ShT managed the ASC preparation. SaT supervised the clinical study. All the authors have approved the manuscript. TS advised the study design, interpreted the data, and revised the draft.

## Ethical approval

Ethical approval for the study protocol was obtained by the institutional review board (approval number: 2018100NI, 2023392NI).

## Funding

This study received no funding support.

## Declaration of competing interest

All authors certify that they have no affiliations with or involvement in any organization or entity with any financial interest or nonfinancial interest in the subject matter or materials discussed in this manuscript.
